# A meta-analysis and systematic review of single vs. multimodal neuroimaging techniques in the classification of psychosis

**DOI:** 10.1038/s41380-023-02195-9

**Published:** 2023-08-10

**Authors:** Alexis Porter, Sihan Fei, Katherine S. F. Damme, Robin Nusslock, Caterina Gratton, Vijay A. Mittal

**Affiliations:** 1 Department of Psychology, Northwestern University, Evanston, IL USA; 2 Institute for Innovations in Developmental Sciences, Northwestern University, Evanston and Chicago, IL USA; 3 Department of Psychology, Florida State University, Tallahassee, FL USA; 4 Department of Psychiatry, Northwestern University, Chicago, IL USA; 5 Medical Social Sciences, Northwestern University, Chicago, IL USA; 6 Institute for Policy Research, Northwestern University, Chicago, IL USA

**Keywords:** Schizophrenia, Neuroscience

## Abstract

**Background:**

Psychotic disorders are characterized by structural and functional abnormalities in brain networks. Neuroimaging techniques map and characterize such abnormalities using unique features (e.g., structural integrity, coactivation). However, it is unclear if a specific method, or a combination of modalities, is particularly effective in identifying differences in brain networks of someone with a psychotic disorder.

**Methods:**

A systematic meta-analysis evaluated machine learning classification of schizophrenia spectrum disorders in comparison to healthy control participants using various neuroimaging modalities (i.e., T1-weighted imaging (T1), diffusion tensor imaging (DTI), resting state functional connectivity (rs-FC), or some combination (multimodal)). Criteria for manuscript inclusion included whole-brain analyses and cross-validation to provide a complete picture regarding the predictive ability of large-scale brain systems in psychosis. For this meta-analysis, we searched Ovid MEDLINE, PubMed, PsychInfo, Google Scholar, and Web of Science published between inception and March 13th 2023. Prediction results were averaged for studies using the same dataset, but parallel analyses were run that included studies with pooled sample across many datasets. We assessed bias through funnel plot asymmetry. A bivariate regression model determined whether differences in imaging modality, demographics, and preprocessing methods moderated classification. Separate models were run for studies with internal prediction (via cross-validation) and external prediction.

**Results:**

93 studies were identified for quantitative review (30 T1, 9 DTI, 40 rs-FC, and 14 multimodal). As a whole, all modalities reliably differentiated those with schizophrenia spectrum disorders from controls (OR = 2.64 (95%CI = 2.33 to 2.95)). However, classification was relatively similar across modalities: no differences were seen across modalities in the classification of independent internal data, and a small advantage was seen for rs-FC studies relative to T1 studies in classification in external datasets. We found large amounts of heterogeneity across results resulting in significant signs of bias in funnel plots and Egger’s tests. Results remained similar, however, when studies were restricted to those with less heterogeneity, with continued small advantages for rs-FC relative to structural measures. Notably, in all cases, no significant differences were seen between multimodal and unimodal approaches, with rs-FC and unimodal studies reporting largely overlapping classification performance. Differences in demographics and analysis or denoising were not associated with changes in classification scores.

**Conclusions:**

The results of this study suggest that neuroimaging approaches have promise in the classification of psychosis. Interestingly, at present most modalities perform similarly in the classification of psychosis, with slight advantages for rs-FC relative to structural modalities in some specific cases. Notably, results differed substantially across studies, with suggestions of biased effect sizes, particularly highlighting the need for more studies using external prediction and large sample sizes. Adopting more rigorous and systematized standards will add significant value toward understanding and treating this critical population.

## Introduction

Psychosis is a devastating and heterogeneous disorder with a poorly understood etiology [1–5]. Psychosis symptoms are thought to emerge from network-level abnormalities within the brain as opposed to disruptions in one discrete location [6–12]. Consistent with the neurodevelopmental theories and stress diathesis models of psychosis, whole-brain structural abnormalities such as impaired myelination [13–15], and accelerated demyelination have been linked with symptom severity and deficits in cognitive function [16, 17]. There are signs of progressive degeneration of other structural measures such as cortical thickness [18] and gray matter volume [19] linked to psychosis. Psychotic disorders have also been characterized as a disruption in the functional communication between brain regions [20, 21] and alterations in the functional strength of connections [22, 23]. These findings suggest that psychosis is characterized by a combination of structural and functional dysfunction across distributed brain systems [8, 23–25].

Non-invasive neuroimaging methods can be used to measure structural (T1-weighted imaging, diffusion imaging) and functional (resting-state functional connectivity) brain networks. Given the link between psychosis and brain system dysfunction, one may ask which specific neuroimaging modalities are best suited for diagnostic purposes, or if a combination of multiple modalities would allow a more holistic and accurate classification of the disorder. While a number of studies have begun to probe this question, this idea has not been tested in a systematic review. This meta-analytic study was designed to directly compare neuroimaging methods (T1-weighted imaging, diffusion imaging, and resting-state functional connectivity) and their combination (multimodal approaches) in their ability to classify psychosis from healthy controls using machine learning data from whole-brain networks. Additionally, this review evaluated whether various statistical, methodological, and demographic information had any moderating effects on classification.

In psychosis, a reduction in gray matter volume and enlargement of the ventricles has been reported through the use of T1-weighted imaging (abbreviated as ‘T1’ in this manuscript) [26]. Although, this may be due to neurotoxic effects related to medications [27], some evidence suggests volumetric differences are present in never medicated and first-episode patients [28]. This would suggest that gray matter abnormalities may be a risk factor leading up to the onset of psychosis or primary aspects of its etiology. Reductions in gray matter can vary with time and are not always consistent across people [19]. Prior work has demonstrated that gray matter cortical thickness declines with age in participants with psychosis at a higher rate compared to controls, particularly in regions important for cognitive function such as inferior frontal cortex, anterior cingulate cortex, and lateral temporal cortex (for review see [18]).

White matter abnormalities such as decreased expression of oligodendrocytes have been associated with psychosis [29, 30]. Diffusion tensor imaging (DTI) is a non-invasive measure of the myelin integrity of underlying white matter [31–33]. Researchers have found that measures of white matter integrity decrease at higher rates in psychosis compared to controls across the lifespan [16, 17]. The development of myelination also tends to proceed and co-occur with the emergence of symptoms of psychosis during the adolescent time period [34]. This work provides evidence of developmental abnormalities that might be linked with psychosis-specific accelerated aging of white matter pathways. However, the location of disruption in the integrity of white matter pathways has remained inconsistent [35, 36]. A recent meta-analytic study aimed at evaluating white matter integrity in high-risk individuals found significant variation in the integrity of white matter pathways across large tracts such as superior longitudinal fasciculus, inferior longitudinal fasciculus, and inferior fronto-occipital fasciculus [35]. These abnormalities tended to vary across study design and have not been consistently linked to variation in symptom severity.

Resting-state functional connectivity (rs-FC) is a non-invasive way to evaluate large-sale functional networks across brain regions. Changes in these networks may be associated with genetic, cognitive, and developmental factors of psychosis. These functional changes have been found to be more closely linked to the expression of behavioral symptoms used in diagnosis [21, 37]. Rs-FC alterations in networks related to higher-order processes such as attention and executive control [21, 38–43] have particularly been highlighted in individuals with psychosis. However, these results are often inconsistent in the direction and location of dysfunction [23, 44]. The inconsistency in the rs-FC literature could be due to individual variation in clinical characteristics: researchers that have evaluated schizophrenia from an individual-specific approach have identified key characteristics linked to symptoms and behavior [45–48]. After accounting for these variations, rs-FC may serve as a key factor in distinguishing characteristics that are specific to psychosis in machine learning classification.

The neuroimaging approaches described above have helped to uncover key neurobiological associations of psychosis. However, there are limitations in each non-invasive method for measuring brain systems [49]. One potential solution is to use a multimodal approach in which different imaging modalities are combined. This multimodal approach may bridge the relationship between gray matter, white matter, and functional features of brain networks that would otherwise be lost when evaluating a single modality. Prior work has suggested that using multiple imaging modalities provides a sensitive approach to identify converging areas of dysfunction in schizophrenia [50–52].

To determine the utility of each method in understanding neurobiological features of schizophrenia, we focus here on machine learning approaches. These methods can use multivariate information to identify subtle variations in the brain that may not otherwise be captured using standard univariate methods [53, 54]. Imaging modalities can also be used as features to classify various forms of psychiatric disorders [55]. However, there are several important factors to consider when using machine learning methods with neuroimaging data including improper cross-validation [56], small sample sizes [37] and physiological artifacts [57–60] that are known to produce inflated or misrepresented classification results.

Here, we completed a systematic review and meta-analysis to determine to what extent neuroimaging methods can classify individuals with psychosis. Specifically, we asked whether any method (or their combination) outperforms others in the ability to distinguish participants diagnosed with a schizophrenia spectrum disorder from healthy controls in the context of machine learning classification. We used a bivariate random-effects model assessing the sensitivity and specificity in each study [61]. To reduce the potential for inflated results, we opted for a strict set of criteria for manuscript extraction including cross-validation. Additionally, we evaluated whether other variables moderate the metrics associated with classification such as preprocessing technique, statistical methods, sample size, and participant characteristics.

## Methods

### Overview

This meta-analysis was conducted following the preferred reporting guidelines for systematic reviews and meta-analysis (PRISMA) [62, 63]. Search criteria limited the analysis to studies that applied classification algorithms to predict clinical status in psychosis participants who met criteria for subtypes within schizophrenia spectrum disorders relative to healthy controls (psychosis v. healthy control). This meta-analysis includes estimates of sensitivity and specificity as calculated based on confusion matrices. A bivariate approach and hierarchical summary receiver operating characteristics (ROC) model were used to estimate sensitivity and specificity across studies. Additionally, we conducted a meta-regression analysis to examine differences between datasets that may contribute to variability found between imaging subgroups (e.g., participant characteristics, statistical methods, and quality of preprocessing methods).

### Search strategy

We searched databases Ovid MEDLINE, PubMed, PsychInfo, Google Scholar, and Web of Science for relevant, peer-reviewed publications. Databases were searched from inception until March 13th 2023. Titles and abstracts were searched using the following keywords: (Schizo* or psychosis or psychotic) AND/OR (DTI or DSI or white matter or fractional anisotropy or FA) AND/OR (fMRI or functional connectivity or network or resting state or rsfMRI or circuit) AND/OR (structural or T1 or anatomical) AND (support vector or SVM or classification or categorization or machine learning). We included advanced search terms to only evaluate studies written in English that included human subjects. In the case of insufficient data, authors were contacted via email to provide additional information.

### Study selection

All titles and abstracts of identified publications were screened by authors A.P. and S.F. for eligibility. Articles had to meet the following inclusion criteria: (1) studies had to apply a machine learning classification model to predict clinical status using neuroimaging data as features. (2) Studies were required to have some form of cross-validation (e.g., leave-one-out, kfolds, test-train split) or external dataset validation. Results from internal cross-validation and external dataset validation were separated in analyses. (3) Clinical participants had to meet a diagnosis for a psychotic disorder following the diagnostic statistics manual (DSM) or the international classification of diseases (ICD). This included first-episode psychosis, first-episode schizophrenia, schizophrenia, schizophrenia with comorbidity, and schizophrenia spectrum disorder. (4) Given our focus on large-scale brain systems, we restricted ourselves to studies that included whole-brain analyses, excluding those focused on single regions or networks. All potential studies were carefully screened for review. If a study included both region-specific and whole-brain analysis, then the whole-brain results were kept for reporting. (5) Classification was based on at least one of the following imaging types: DTI, rs-FC, T1, or some combination. As we were interested in focusing on intrinsic brain networks rather than task modulations, task-based FC studies and dynamic FC studies were excluded from this analysis. Use of a simulated or synthetically created dataset were also grounds for exclusion. Each neuroimaging type required at least 5 studies to be used for formal meta-analysis [64]. All neuroimaging types examined reached this criterion.

Publications were excluded based on the following criteria: (1) failure to obtain full text of manuscript online or upon request from authors, (2) insufficient information for quantitative extraction, (3) non- or limited- peer-reviewed publications, including conference proceeding abstracts, and (4) intervention-based study designs. We included multiple studies reporting on the same original dataset. All studies were kept for qualitative analysis. To reduce the likelihood of overfitting due to non-independence across results and dataset decay [65], we calculated the mean classification metrics across studies that used the same dataset and included this combined result in our quantitative analysis. Overfitting from repeated re-use of the same public dataset can lead to minimal increases in prediction of unseen or independent data across different machine learning classifiers within clinical populations [66].

### Data extraction

Our primary estimates extracted from each study included sensitivity, a measurement used to assess the model’s ability to accurately predict a psychosis participant correctly, and specificity, which measures the probability of accurately predicting a healthy control. Sensitivity is derived as the number of psychosis participants correctly identified by the classifier divided by the sum of all psychosis participants in the sample. Similarly, specificity is calculated as the number of healthy control participants correctly identified by the classifier divided by the sum of all healthy control participants. From this measurement we can derive the false positive rate (FPR = 1 - specificity); this measurement specifies the probability of incorrectly labeling a healthy control as someone with psychosis.

We also collected the following information: year of publication, participants characteristics such as group size, age, gender, antipsychotic medications (as converted to chlorpromazine (CPZ) equivalents), illness duration in months, handedness, nicotine use, symptom severity as measured by the positive and negative syndrome scale (PANSS [67]), and analysis characteristics such as dataset origin if using publicly available data, neuroimaging modality type (T1, DTI, rs-FC, or multimodal), classification method (e.g., support vector, ridge regression, decision tree), cross-validation procedure (e.g., leave one out, kfold, train test split), and number of features. If studies reported performance from multiple predictive models all measures were initially extracted. In the case of more than one statistical model, the sensitivity and specificity scores averaged across all models were used for the quantitative analysis.

Prediction results were classified as based on internal prediction (within dataset cross-validation) or external prediction (validation in a new dataset); these were used for separate quantitative analyses. When manuscripts incorporated both an internal and an external dataset for, both sets of performance measures were kept.

If multiple different studies used the same dataset for analysis, we recorded the sensitivity and specificity values for each study separately for reporting purposes and qualitative review, and included the mean across studies with that dataset for quantitative analysis to reduce the risk of overfitting (as discussed above). In the case of studies including several different datasets for model training, manuscripts were excluded if datasets were already in use among other studies, otherwise, the average pooled result was included for the quantitative analysis. When examining studies involving more than one clinical subgroup or first-degree relative we extracted classification measures specifically for participants diagnosed with psychosis and healthy control groups for quantitative analysis.

### Statistical analysis and assessment of bias

Cochrane’s Q and I^2^ tests of heterogeneity were used to determine significant differences between studies and modalities (T1, DTI, rs-FC, multimodal) using a random effects model [68]. Q is used to assess that the proportion of successful classification is equal for all groups (healthy control and psychosis). Q is defined as the weighted sum of squared deviations from individual study effects (log odds ratio) against the pooled effect across studies. To determine if there is heterogeneity within and across imaging groups we formally test whether Q follows a chi-squared distribution with k-1 degrees of freedom. If the null hypothesis is rejected (*p* < 0.05) heterogeneity is likely present. Heterogeneity can also be measured using I^2^. This measure describes the amount of variation present across studies [69, 70]. This procedure is calculated as a percentage of Q minus the degrees of freedom divided by Q. Heterogeneity was operationalized as small (I^2^ = 25%), moderate (I^2^ = 50%), or large (I^2^ = 75%) [71].

To evaluate the potential for systematic bias in published results, several analyses were conducted. First, we created funnel plots for visual inspection of effect sizes for each imaging modality. This figure plots the effect estimates from each study against the standard error of effect estimates. This plot is used to evaluate the variation in classifying psychosis while accounting for sample size. If published effects are unbiased, then one should assume that no correlation exists between standard error and effect estimates after accounting for sample size heterogeneity across studies [72]. However, a correlation between standard error and effect estimates (seen as an asymmetry in the funnel plots), would suggest that there is some form of bias across studies that is not due to random sampling variation. Bias can be due to a number of factors such as publication bias, selective reporting, poor methodological design, and high heterogeneity [72]. Publication bias is just one of many potential reasons for asymmetry and it is impossible to know the precise mechanisms of asymmetry.

To formally test for funnel plot asymmetry, we conducted an Egger’s regression test [73] and an alternative test called Peter’s test [74]. Egger’s test is a linear regression model of the estimates (log diagnostic odds ratio) on their standard errors weighted by their inverse variance. While commonly used, this method can be problematic for assessing log odds ratio based estimates as the standard error is dependent on the size of the odds ratio even in the absence of small study effects [74]. The Egger’s test can produce false positive results when sample sizes across groups (healthy control and psychosis) are not evenly balanced [74]. Peter’s test is an alternative test that instead uses the inverse of the total sample size as the independent variable, thereby accounting for heterogeneity across groups (healthy control and psychosis) without increasing the likelihood for Type I errors.

Two sets of meta-analyses were conducted, one in which all studies were used and one in which a subset of outlier studies was excluded. For these meta-analyses, we implemented a bivariate approach in which sensitivity and specificity scores were log-transformed and combined into a bivariate regression model [61]. This approach is useful to assess diagnostic accuracy by accounting for biases in sensitivity and specificity [75]. Due to variation in modeling methods and specific cutoff thresholds for sensitivity and specificity, a random-effects model was applied. Each study was weighted based on sample size to account for variation in effect size. Statistical analyses were conducted using R [76]. To evaluate sensitivity and specificity values and conduct a bivariate meta-regression model to examine moderating effects of statistical methods, participant characteristics, and preprocessing method (when applicable) on the pooled estimates the packages *mada* [77] and *metafor* [78] were implemented. To reduce heterogeneity across studies, analyses were separated into internal prediction (via cross-validation in the same dataset), and external prediction (in a new dataset). A significant main effect was determined based on the use of a likelihood ratio test comparing the derived model to a null model. Based on this result, follow-up pairwise comparisons are conducted to determining the level of significant across factors (e.g., comparing each imaging type).

### Quality assessment of rs-FC preprocessing

To evaluate how denoising influences classification, we derived a quality measure of the denoising procedure at removing motion artifacts (Table 1) and related this rating to classification performance. This analysis was limited to rs-FC datasets, as other modalities did not include as many denoising procedures (Supplemental Table 1–4). The rating was based on results reported by Ciric and colleagues [57], which systematically compared the ability of different processing pipelines to remove motion biases in rs-FC analyses. The score was based on two criteria that measured the two major influences of motion on functional connectivity [57, 59]: (1) the total percent of edges related to head motion in each strategy (Fig. 2 in [57]) and (2) the distance-dependent influences of head motion on functional connectivity (Fig. 4 in [57]). The final score was weighted such that up to 75% of the final score was based on the first criteria and up to 25% of the final score was based on the second criteria (to reflect the relative difference in their impact on functional connectivity values [57]). Note that additional tests were also conducted on each criteria separately.

**Table 1:** Overview of scaling method used for assessing quality of preprocessing methods.

Good performance 1	Moderate performance 2	Moderately poor performance 3	Poor performance 4	Extreme Concern 5
Percent of Edges related to Motion				
(0–10% edges correlated with motion)	(10–20%)	(20–30%)	(30–40%)	(> 40%)
36P+spkreg 36P+despike 36P 36P+scrub	ICA+GSR 9P aCompCor	ICA	wmMean	2P 6P 24P wmLocal tCompCor
Distance dependence of motion effects				
QC-FC corr r > −0.15	r = −0.15 to −0.2	r = −0.2 to −0.25	r = −0.25 to −0.3	r < −0.3
ICA 36P+scrub	ICA+GSR wmLocal	24P 6P 36P+spkreg wmMean 36P+despike	acompcor 2P	9P tcompcor 36P

Percent of edges related to motion and distance dependence of motion effects are described in detail elsewhere [57]. 36 *P* = nuisance regressors included 6 motion estimates, mean white matter (WM), mean cerebral spinal fluid (CSF), and mean global signal (GS), along with the derivatives, quadratic terms and squares of these signals [171]. 36 P+despike = includes 36 regressors as described above, with despiking removal of high motion frames [194]. 36 P+spkreg = includes 36 regressors with spike regression of high motion frames [171]. 36 P+scrub = 36 parameters and motion scrubbing of high motion frames [59]. Scrubbing high motion frames were defined using framewise displacement (FD), computed as the sum of the absolute values of the derivatives of translational and rotational motion estimates. FD > .2 mm was flagged as high motion. 2 *P* = nuisance regression includes mean WM and mean CSF.

6 *P* = nuisance regression only includes 6 motion estimates from realignment. 9 P + GSR = nuisance regression includes 6 motion estimates, mean WM, mean CSF, and mean GSR [195, 196]. 24 *P* = nuisance regression includes 6 motion estimates, their temporal derivatives and quadratic expansion terms [197]. aCompCor = nuisance regression includes 5 principal components each from the WM and CSF, in addition to 6 motion parameters and their temporal derivatives [198]. tCompCor = nuisance regression includes 6 principal components from voxels with high variance over time [199]. wmLocal = nuisance regression includes a voxelwise localized WM regressor in addition to 6 motion parameters, and their temporal derivatives and despiking [200]. wmMean = nuisance regression includes mean WM in addition to 6 motion parameters and their temporal derivatives and despiking [200]. ICA = independent component analysis, removal of motion-related variance components from the BOLD data including mean WM and CSF regressors [201].

For the first criteria, each processing strategy was given a score scaled 1–5 with 1=good performance at removing motion artifacts in rs-FC (i.e., 0–10% edges contaminated by motion), 2=moderate performance (10–20%), 3=moderately poor performance (20–30% edges contaminated by motion), 4=poor performance (30–40% contaminated by motion), and 5=extreme contamination (>40% edges contaminated by motion). Manuscripts that included an additional step evaluating or excluding subjects based on framewise displacement (FD), resulted in one point subtracted off the initial edge score (i.e., subject removal, reporting of mean FD, group-related differences in FD, or any other mitigation strategy [60]).

For the second criteria the score was based on the magnitude of distance-dependent motion artifacts with 1 = good performance of minimizing distance dependence (r > −0.15), 2=moderate performance (r = −0.15 to −0.2), 3 = moderately poor performance (r = −0.2 to −0.25), 4=poor performance (r = −0.25 to −0.3), and 5=extreme contamination (r < −0.3). Each processing strategy was scored based on these criteria as shown in Table 1. Note that for the purposes of scoring, all ICA methods were grouped with ICA-AROMA as the closest comparator; other methods were also grouped with their closest fitting denoising approach. A composite score representing the quality of the denoising pipeline was generated with a 75% weighting from the edges contamination measure and a 25% weighting from the distance-dependent influence of motion.

Any manuscript using rs-FC features for classification was used in this analysis. Each manuscript’s denoising methods were scored by 3 independent reviewers (authors A.P., S.F., and C.G.), and that assigned value was used for the quantitative analysis. Inter-rater reliability was high across reviewers (100%).

## Results

**Fig. 1:**
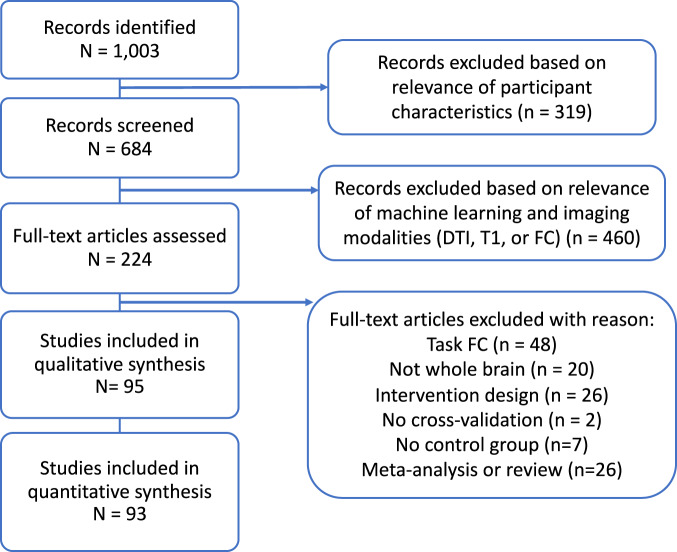
Flow diagram mapping the selection of studies for the classification of psychosis-based disorders from neuroimaging modalities, following PRISMA guidelines [64, 65]. Full inclusion and exclusion criteria are listed in *Methods*.

The initial search yielded a total of 1003 manuscripts; 684 remained after removing manuscripts that did not involve psychosis-based disorders. Articles were then restricted based on those that included machine learning classification based on the selected MRI imaging modalities (T1, DTI, rs-FC). This led to 224 manuscripts that were analyzed for further review. Full-text publications were assessed for eligibility and after full text review 95 articles were retained for qualitative review and 93 for quantitative review (for a detailed breakdown of inclusion see Fig. 1 and Supplemental Table 1–4)). Articles were removed from analysis for the following reasons: FC derived from tasks rather than rest, region/network specific analysis (not whole brain), intervention or longitudinal design, lack of cross-validation, lack of healthy controls, and review or meta-analysis manuscripts.

Results were separated for studies using internal validation (cross-validation within the same dataset) vs. external validation (validation within a new independent dataset). We focus first on reporting analyses from results of the larger internal validation group. This initial analysis consisted of 28 T1 [79–109], 9 DTI [48, 110–117], 38 rs-FC [48, 118–156], and 14 multimodal [157–170] subgroups. From this sample, we identified 21 manuscripts that used the overlapping datasets for classification [86, 97, 102, 105–109, 116, 138–140, 142–154, 156, 166–169, 171]. To decrease the risk of inflation from non-independence and overfitting from dataset decay [70], we calculated the mean sensitivity and specificity scores across all studies that used the same dataset with the same imaging modality for the primary analyses reported in this manuscript. In the case that the same dataset was used with a different imaging modality for classification, we calculated the average measures per modality. This resulted in the inclusion of 3 rs-FC reports (using the W. China [172], Cobre [173], and NAMIC [174] datasets), 2 T1 (BRNO [106], Cobre [173]), and 1 multimodal (Cobre [173]). The reports from each analysis and the represented average are shown in detail in Supplemental Fig. 1.

However, we also conducted a parallel set of analyses in which manuscripts that pooled information across overlapping datasets were included separately, in order to provide information based on larger, better powered studies. These manuscripts are also included Supplemental Table 1–4 and results from this parallel analysis are shown in Supplemental Fig. 2 and reported in the results sections below.

### All tested imaging modalities have a moderate ability to predict psychosis

**Fig. 2:**
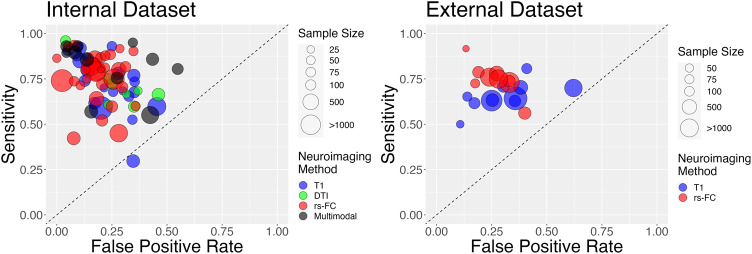
Sensitivity plots of psychosis classification performance when tested on independent internal (left; i.e.., cross-validation) and external (right) datasets. Sensitivity and specificity scores were derived using data from the classifier in each manuscript. All manuscripts were able to reliably differentiate participants with psychosis from healthy controls independent of neuroimaging type aside from [169]. The size of points is scaled according to sample size and modality of analysis is shown in various colors.

**Fig. 3:**
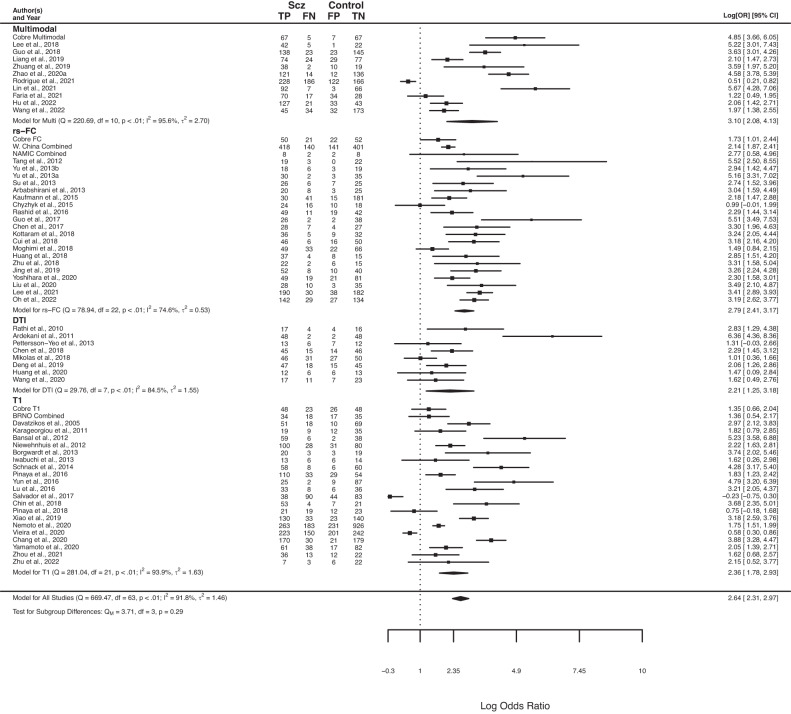
Forest plots for log diagnostic odds ratio, separated by imaging modality. Summary forest plot for log diagnostic odds ratio for all imaging modalities presented at the bottom of the plot. Multimodal: classification that used at least two of the following: rs-FC, T1, and/or DTI as features. RS-FC: resting state functional connectivity. DTI: diffusion tensor imaging. T1: T1 weighted imaging. The point size of squares and polygons are a function of the precision of the estimates.

Aside from one T1 study [104] we found that all studies, independent of sample size, were able to reliably differentiate psychosis from healthy controls for all imaging modalities (Fig. 2, Fig. 3). Average sensitivity and specificity measures were modest across all imaging groups (T1: sensitivity = 0.73 + /−0.15, specificity = 0.77 + /−0.11; DTI: sensitivity=0.71 + /−0.11, specificity = 0.73 + /−0.12; rs-FC: sensitivity=0.76 + /−0.13; specificity=0.81 + /−0.09; Multimodal: sensitivity=0.81 + /−0.14, specificity=0.79 + /−0.17). Similar results were seen when larger-sample studies with pooled datasets were included in analysis (Supplemental Fig. 2).

We next examined whether specific modalities were better able to classify psychosis. Using a bivariate analysis, we did not find a significant difference in internal classification performance based on imaging modality (*p* > 0.05; Fig. 3). Including pooled datasets resulted in a similar finding (*p* > 0.05; Supplemental Fig. 2). These results suggest that, based on methods in the current literature, combining multiple neuroimaging methods to track psychosis does not provide any major advantage relative to single imaging modalities on average.

We also conducted a separate bivariate analysis using studies that provided classification performance in an external dataset (*N* = 21). Due to the small number of studies, we were limited to quantitatively contrasting the use of T1 (14) or FC (12) imaging modalities. As might be expected, average sensitivity and specificity values were slightly lower compared to the internal results (Fig. 2; T1: sensitivity=0.66 + /− 0.07, specificity = 0.69 + /− 0.14; rs-FC: sensitivity = 0.75 + /− 0.09, specificity = 0.74 + /− 0.09). In this analysis, we did not find a difference in performance by imaging modality. However, when pooled datasets were included we found a statistically significant association between imaging modality and classification performance in external samples (Sensitivity z = 0.34; *p* = 0.003, Supplemental Fig. 3). This result indicates that rs-FC outperforms T1-based classification of psychosis in external datasets when large pooled datasets are used.

Notably, a close examination of these results indicates that there is substantial heterogeneity in classification performance across studies. We next analyze this heterogeneity in more detail, and ask whether there is bias in reported effects and how this bias affects classification performance.

### Addressing heterogeneity reveals differences in psychosis prediction across imaging modalities

Neuroimaging subgroups appeared to have high amounts of variability as measured by Cochrane’s Q (Χ^2^ = 1,004.06, *p* < 0.0001). We asked whether this variability in reported effects exhibited any evidence of bias. This was evaluated by examining signs of funnel plot asymmetry, Egger’s test [73] and an alternative test [74]. Funnel plot asymmetry can indicate a bias of reported effect estimates and standard error after accounting for sample size heterogeneity.

**Fig. 4:**
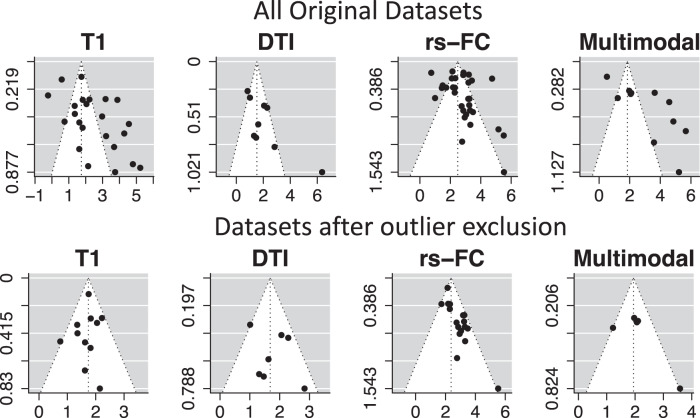
Funnel plots with psychosis classification performance, broken down into subplots based on neuroimaging modality. Note: these results are based on internal validation only, given the higher number of studies in this domain. The top row shows funnel plots for all original internal datasets, while the bottom row shows funnel plots after outlier exclusion. Each plot is centered on a fixed effect summary estimate, the outer dashed lines indicate the 95% confidence interval of the fixed effect estimates. Symmetry is apparent when all studies are randomly dispersed around the dashed vertical line. In contrast, each imaging group showed signs of funnel plot asymmetry. These observations were confirmed by formally testing the correlation between study size and effect estimates using the Egger’s test (*p* < .05).

Funnel plots and Egger’s test produced evidence of asymmetry in reported effects for all modalities (*p* < 0.05; Fig. 4), suggesting that reported effects may be biased. Bias can occur due to publication bias, selective reporting, poor methodological design, and high heterogeneity of findings [72]. However, use of the Peter’s test resulted in no significant evidence of funnel plot asymmetry (*p* > 0.1). These conflicting results are likely due to the large heterogeneity across studies and use of log odds ratio estimates (as described in more detail in ref. [74]).

To address potential confounding results due to funnel plot asymmetry, we conducted an additional set of analyses similar to our primary ones, but after removing outlier studies. Outlier studies were identified as lying outside the 95% confidence interval of the pooled effect among each imaging group. This resulted in a final sample of 11 T1, 8 DTI, 21 rs-FC, and 5 multimodal internally-validated studies (Fig. 4, highlighted in Supplemental Table 1–4). After outlier exclusion, a dip test was conducted and did not reveal significant signs of non-unimodal distribution within each imaging group (*p* > 0.1).

**Fig. 5:**
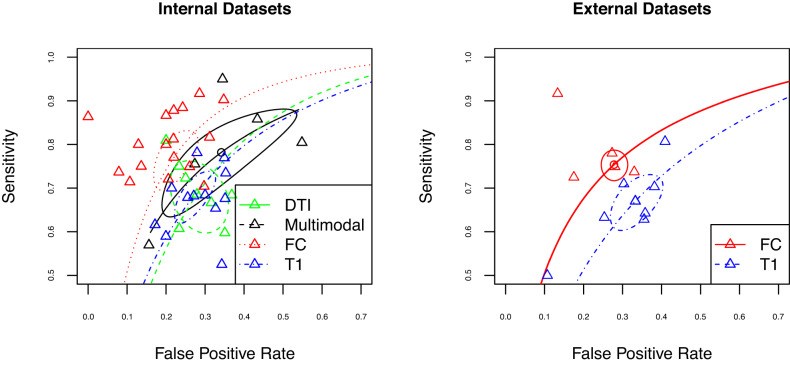
Sensitivity plots of psychosis classification performance after the exclusion of outlier studies, when tested on independent internal (left; i.e.., cross-validation) and external (right) datasets. Colors represent different imaging modalities; estimated summary receiver operating curves are shown overlaid for each of the imaging modalities with respective confidence interval regions surrounding mean sensitivity and specificity values. No significant differences were found for classification of internal datasets . When external datasets were used for classification, rs-FC studies (red) have a significant advantage in classification relative to T1 studies (blue).

In this reduced set of manuscripts, we continued to observe similar classification performance across modalities, with no significant difference in psychosis classification across imaging modalities when classification was tested on independent internal datasets (i.e., via cross-validation; *p* > 0.05; Fig. 5 left). If larger pooled datasets were included in the meta-analysis, studies based on rs-FC were associated with a significant decrease in false positive classification rates compared to studies based on DTI (Supp. Fig. 3; z = −0.58, *p*(FDR) = 0.01).

When classification was tested on external datasets, we also found signs of funnel plot asymmetry (*p* < 0.05). After outlier exclusion, there was still a significant difference between imaging modality and classification, with studies using rs-FC resulting in higher sensitivity rates (z = 0.42, *p*(FDR) < 0.001) compared to T1 imaging (Fig. 5, right). This effect remained consistent when including pooled datasets (z = 0.39; *p*(FDR) < 0.001; Supplemental Fig. 3).

No other differences were present across modalities. Notably, in all versions of analyses, rs-FC studies performed similarly to multimodal studies, and multimodal studies did not outperform other imaging modalities in their prediction of psychosis. These findings suggest that, with the current literature, most imaging modalities perform similarly in psychosis classification, without major advantages for multimodal methods relative to unimodal methods (although rs-FC shows some slight advantages over structural-based approaches).

### Classification performance is not associated with demographic or analysis covariates

In our final set of analyses, we investigated different potential sources of variation in prediction results (for a detailed overview see Supplemental Table 5). First, we examined participant characteristics: age, gender, CPZ equivalents, illness duration, and PANSS (we did not obtain enough studies that reported handedness or nicotine use to conduct a bivariate model assessing these participant characteristics). When conducting a bivariate analysis using all studies independent of imaging modality, we did not find a moderating effect on sensitivity or specificity measures for any of these factors (*p* > 0.1).

Next, we examined analysis characteristics. We did not find moderating effects based on classification method (e.g., support vector, ridge, decision tree), deep vs. non-deep methods, cross-validation scheme (e.g., leave one out, test train split, kfold), feature size, or publication year (*p* > 0.1)(see Supplemental Fig. 4). We also did not find a significant main effect based on sample size (*p* > 0.1).

Head motion is a major confound in neuroimaging analyses [57–59, 175]. Therefore, we conducted an additional analysis to evaluate whether variance in effects sizes could be related to denoising methods designed to reduce the influence of head motion. This analysis was performed with a subset of studies that were either primarily rs-FC based or multimodal (rs-FC + other methods) (*N* = 50). We examined the influence of motion related artifacts on rs-FC effect sizes using a manually derived quality assessment score (as described in *Quality assessment of rs-FC preprocessing*). We found that the total quality assessment measure did not have an effect on classification (X^2^(2,50) = 0.34, *p* = 0.8). This result suggests that motion artifacts were not a major driver of classification performance. However, it is important to note that the majority of rs-FC studies used similar motion denoising techniques.

## Discussion

We conducted a meta-analysis to test whether there are advantages to combining neuroimaging modalities for classification of individuals with psychosis. This analysis yielded several surprising and important findings. First, we found that all neuroimaging modalities examined (T1, DTI, rs-FC, multimodal) were able to classify individuals with psychosis from healthy controls. Second, we only found limited differences across modalities, primarily in advantages of rs-FC relative to T1 in classification performance in external datasets. Third, there was significant evidence for heterogeneity across studies. The reported effect sizes within each imaging group appear asymmetric, suggesting that systematic bias may be present in past reports of the classification of psychosis. When studies outside of the expected confidence bounds were removed, we continued to detect only limited differences across modalities, primarily associated with rs-FC approaches relative to structural imaging methods (DTI when classification was tested with internal datasets; T1 when classification was tested with external datasets). Notably, across all analyses, no difference was seen between multimodal and rs-FC approaches, which had largely overlapping classification distributions. We further discuss the implications of these findings and provide suggestions for improvements in future studies below.

Given that the extant of psychosis literature has identified changes in both the function and structure of whole brain networks, it is, perhaps, surprising that we did not see large improvements in classification when using multimodal methods compared to all unimodal methods. Interestingly, in literature predicting behavioral variables from neuroimaging, recent papers have also found that multimodal methods do not provide an increase in performance relative to single modalities [176, 177]. This finding is in direct contrast to prior work arguing for the advantages of multimodal approaches (for review see [178]). This may be specific to these particular classification cases, to limitations in current methods in merging multimodal results, or due to individual differences in the function and structure of networks which introduces noise during classification and does not aid in prediction [179, 180].

When our analyses were restricted to remove outlier studies, we found evidence that rs-FC approaches statistically outperform structural (DTI for internal and T1 for external) studies in classifying psychosis. This suggests that heterogeneity in study results (discussed further below) may also limit our ability to detect modality-based effects. It is interesting that even in this circumstance, multimodal approaches did not differ from functional (rs-FC) approaches and had largely overlapping distributions. It is possible that different advantages between the modalities will be identified that pertain to specific questions and sub-populations, and that differences across modalities will be enhanced with additional methodological and analytical development. While the use of multimodal methods has gained popularity and can, at times, produce advantages to predicting psychopathology and cognition [178, 181], these differences in accuracy may not fully capture the inherent variation in sensitivity and specificity that was found across studies. We look forward to seeing additional methodological development and larger studies in this area that will help expand knowledge in this domain.

Results from Egger’s test and the funnel plots demonstrated asymmetry in effect sizes among reported studies. This finding suggests that the ability to predict psychosis is negatively correlated with study precision (as measured by variance per participant group). Asymmetry in reported effects is usually evidence for systematic bias, independent of random sampling variance. This effect may be due to selective biases in reporting, poor methodological design, or inflated effects from small sample sizes. Publication bias, or the selective reporting of results that produce a significant effect, is one potential interpretation of why asymmetry was present in this analysis. Notably, however, removing studies outside of the expected confidence interval bound from our funnel plot analysis did not substantially change results regarding classification performance across modalities, aside from revealing a difference between rs-FC and DTI studies in prediction of internal datasets. We are hopeful that the increase in predictive modeling and popularity in preregistration of projects (e.g. Open Science Framework) will help reduce the effect of systematic bias in publication over time.

It is important to highlight that the Egger’s Test can produce false positive results when sample sizes across participant groups are not evenly balanced [74]. Our meta-analysis included manuscripts with a wide range of group-level sample sizes that were not always balanced across participant groups (*n* = 10–600 per group). When we conducted an alternative test for asymmetry using Peter’s Test we did not find evidence of funnel plot asymmetry. From this analysis, we conclude that the asymmetry of effect sizes is likely at least in part associated with unbalanced participant groups. Future work should seek to align the sample size of participant groups prior to classifying psychosis.

Recent work has shown that separation of machine learning models based on race, gender, and age results in significant differences in classification performance [182, 183]. These findings indicate that there are biases in classification for certain groups, and that the lack of diversity in samples may lead to poor performance in broader and more diverse samples. We did not see a difference in classification based on age or gender within this meta-analysis (Supplemental Fig. 4), but were likely underpowered for conducting a more rigorous analysis to determine if diversity characteristics had an effect on performance. Future work should evaluate how classification in psychosis varies when a diverse sample of individuals is used.

When evaluating studies that used external datasets for prediction, we found that functional (rs-FC) methods were significantly better at classifying psychosis compared to structural (T1) methods. It is important to note that we were limited to quantitatively contrast the use of T1 or rs-FC imaging modalities due to the small number of studies that use external prediction across other imaging modalities. Future work should place more emphasis on using external datasets to determine the extent to which a model generalizes and to provide an unbiased view of predictive performance across different imaging types. Notably, psychosis classification in external datasets was slightly lower than in internal datasets, suggesting that internal classification is inflating classification prediction abilities.

In addition to sample size, studies over the past decade have reported that head motion can systematically alter rs-FC estimates, and reduction of these biases requires appropriate preprocessing strategies [57–60]. When examining the quality of preprocessing methods in rs-FC we did not find that motion preprocessing impacted classification performance. This result could be due to the limited range of motion denoising methods across the majority of rs-FC based studies. There is substantial evidence that motion, respiration, and other physiological artifacts can significantly bias estimates of rs-FC [57–60]. These biases in rs-FC are of particular concern within psychosis samples [184–186]. Caution should be employed when evaluating classification metrics using rs-FC if preprocessing methods do not properly account for motion. As the field advances in motion filtering techniques, future work will need to reevaluate the effects of motion and classification in the context of psychopathology.

Notably, motion has also been demonstrated to impact DTI and T1 measures and lead to misleading results such as reduced volume and gray matter thickness [187, 188] and distorted measures of FA [189, 190]. Unfortunately, very few DTI and T1 studies addressed motion to a great extent, limiting our ability to analyze whether preprocessing strategies on effect sizes. Future work should evaluate how motion artifacts in these imaging modalities can also influence classification [191].

### Limitations and future directions

The use of neuroimaging-based classification holds considerable promise towards supporting clinicians in the diagnosis of psychopathology. Here, we found that many different neuroimaging methods were able to classify psychosis, but that these methods performed largely similarly, with slight differences observed between functional and structural imaging measures. There are important factors to consider that could influence the outcome of these findings.

Recent work has demonstrated that identification of behavioral phenotypes linked to psychopathology requires very large sample sizes (*N* > 2000) in order to produce replicable results when using rs-FC and structural MRI measures [37]. Studies that met criteria for this meta-analysis varied considerably in sample size (20–1100), but were generally substantially smaller than this recommended size. Due to the limited number of studies available, we were unable to obtain manuscripts that utilized large enough samples and could not formally evaluate how samples larger than 2000 participants performed across imaging modality. As the trend to increase sample size continues, future work should reevaluate whether the combination of neuroimaging modalities provide substantial advantages when sample sizes are sufficiently large.

Notably, several of the largest sample sizes present in classification studies were associated with pooling of (the same) large public datasets. Given their non-independence, and in order to reduce the risk of overfitting and dataset decay [65] for these large datasets, we included all of these results as a single average classification statistic in our primary quantitative analyses. However, this limited our ability to include datasets with increased sample sizes, which is an important limitation in machine learning studies. Therefore, we included a parallel set of analyses (reported in Supp. Fig. 1–3) that conducted meta-analyses with these large pooled datasets included separately. These results paralleled the findings from the primary analyses, indicating that, at present, the use of dataset pooling is not substantially altering findings in psychosis classification.

In our search for large-scale network markers that predict psychosis, we were selective in the studies that met criteria for our analysis, requiring that, for example, they included whole-brain analyses, used cross-validation, and reported classification measures (and for functional connectivity analyses, were based on static rs-FC; for full list of inclusion and exclusion criteria, see *Methods*). We restricted our meta-analysis to static resting-state FC, excluding task-based FC and dynamic approaches (given the wide variation in these, and the desire to measure task/variation effects), and we only included whole brain analyses as opposed to region-specific analyses, as our interest was to evaluate widespread changes in brain networks. These restrictions resulted in a surprisingly large number of rejected studies (*N* = 68). Future work will be needed to systematically contrast different networks/regions and different functional connectivity methods on their ability to classify psychosis; past work has suggested commonalities across these functional network methods [192], but that task effects can also significantly influence prediction [193].

In addition to overlapping datasets, we only included manuscripts that had been peer-reviewed and indexed in PubMed Central or Web of Science. This approach limited our inclusion of conference abstracts, focused on recent advances. A number of abstracts from IEEE conferences [194–199] provide a glimpse into the outlook and growth of advanced modeling techniques for predicting psychosis. Generally, these studies demonstrate similar trends in performance as the studies included in the meta-analysis and highlight the field’s continued increase in evaluating the clinical potential associated with predictive models [200, 201].

Our investigation included results from a range of different machine learning classification methods in the prediction of psychosis, including deep learning (*N* = 23). There has been an exciting increase in analyses that have used the analytical potential of deep learning methods for prediction in psychopathology, often adopting collaborative (team-based) approaches [194, 202, 203]. However, recent research has also highlighted the limitations in deep learning approaches relative to more conventional machine learning models [204–208], including potential for overfitting datasets [66] and the lack of consistent quantitative benefits relative to prior work [209]. Consistent with Eital [209], in our meta-analysis, we did not find any significant differences in classification performance with deep learning methods relative to other machine learning methods (see Supplemental Fig. 4). However, this area of investigation is relatively nascent, and we believe that it will be valuable to continue additional investigations regarding the precision of deep learning methods relative to other algorithms.

Furthermore, it is worth noting that T1 and DTI data can be analyzed through a variety of methods (e.g., T1 can be used to analyze surface area, thickness and volume) that do not measure the same neurobiological underpinnings. Due to the variation in T1 measures used across each study within this meta-analysis, and the relatively small number of studies passing our criteria, we were unable to perform a more sophisticated model comparing each measurement type (surface area, cortical thickness, etc.) as it relates to classification. We look forward to seeing the addition of progressively more studies of each of these types taking a whole-brain approach to allow for the evaluation of many different networks in evaluating psychosis.

Finally, this analysis was restricted to only include classification between psychosis and healthy controls. It is possible that differences in network organization between healthy controls and psychosis are quite large resulting in higher classification than would be expected when evaluating more nuanced comparisons (e.g., schizophrenia vs. bipolar vs depression). The scope of this analysis was focused on the interplay of function and structure in brain networks related to psychosis and did not extend to other disorders. Future work should evaluate how various disorders and comorbidities relate to classification and neuroimaging modalities.

## Conclusions

All imaging techniques were able to classify psychosis from healthy controls. When accounting for variation in funnel plot asymmetry, we found significant evidence that rs-FC methods outperform structural imaging modalities. However, the results did not find significant differences between multimodal and rs-FC, suggesting that rs-FC may provide thorough information for classification. Future work should apply stringent guidelines when evaluating the predictive nature of neuroimaging modalities among psychosis.

### Supplementary information

Supplement

## Data Availability

This review was not registered. The data supporting these findings of this study are available within this article and its supplementary materials.

## Code availability

Availability of code to derive these findings can be found online (https://github.com/GrattonLab/Porter_metaAnalysis2022).
