# Supplementary material for: A meta-analysis and systematic review of single vs. multimodal neuroimaging techniques in the classification of psychosis
Source: Mol Psychiatry. 2023 Aug 10;28(8):3278–92. doi: 10.1038/s41380-023-02195-9 (PMC10618094; doi:10.1038/s41380-023-02195-9)
Supplement: Supplementary file 1 — Supplement [file 41380_2023_2195_MOESM1_ESM.pdf]

Study	Psychosis Sample (N)	Control Sample (N)	Imaging	CV	Statistical method	Sensitivity	Specificity	Age Psychosis	Age Controls	Gender Psychosis (%F)	Gender Controls (%F)	CPZ Equivalent	PANSS Positive	PANSS Negative	PANSS Total	Illness Duration (months)	MRI Scanner	TR/TE (ms)	Feature Size	Diagnosis	Notes
Davatzikos et al., 2005	69	79	RAVENS map	LOO	SVM	0.74	0.87	29.9	28.2	33%	48%	NA	NA	NA	NA	59.4	3T GE Signa	35/6	NA	SCZ	I
Karageorgiou et al., 2011	28	47	GMV	LOO	LDA	0.66	0.74	24.32	26.38	21%	43%	NA	NA	NA	NA	26.4	Siemens 3 T Trio, Siemens 1.5 T Sonata, Siemens 1.5 T Avanto	2530/3.79, 20/6, 12/4.76	NA	SCZ	I MCIC Dataset ○
Bansal et al., 2012	65	40	Surface morphology	LOO+TTS	Hierarchical Clustering	0.91	0.95	42.16	32.42	63%	55%	NA	NA	NA	NA	NA	1.5 T GE Signa LS	24/5	10000	SCZ	I Wexler et al., 2009 Dataset
Niewehnhuis, et al., 2012	155	122	VBM (including striatum) GMD	LOO	SVM	0.72 (I) 0.71 (E)	0.68 (I) 0.7 (E)	27.18	27.18	48%	48%	NA	15.34	18.6	71.56	123.6	5T Phillips NT	30/4.6	157256	SCZ	I/E Boos et al., 2012 Dataset ○
Borgwardt et al., 2013	23	22	RAVENS map	10F	SVM	0.87	0.86	26.78	23	26%	41%	NA	NA	NA	NA	NA	1.5T Siemens Magnetom Vision	9.7/4	410	FEP	I FePsy Dataset
Iwabuchi et al., 2013	19	20	WMV or GMV	LOO	SVM	0.66	0.73	33.2	32	26%	25%	NA	NA	NA	NA	92.4	7T Phillips Achieva	15/5.6	NA	SCZ	I ○
Schnack et al., 2014	46	43	VBM GMD	LOO	SVM	0.69 (I) 0.66 (E)	0.82 (I) 0.85 (E)	31	33.8	28%	51%	NA	NA	NA	NA	86.4	3T Phillips Achieva	10/4.6	NA	SCZ	I/E Scheewe et al., 2012 Dataset
Sabuncu et al., 2015	50	73	VBM	K Fold	SVM	0.6	0.62	34.3	34.1	18%	18%	NA	NA	NA	NA	NA	NA	NA	Varied	SCZ	I Cobre Dataset
Janousova et al., 2016	52	52	GMV, DBM	LOO	SVM	0.73	0.8	23	23	0%	0%	NA	NA	NA	NA	NA	1.5T Siemens Symphony	1700/3.93	2673601	FES	I BRNO dataset
Lu et al., 2016	41	41	TBV or WMV and GMV--AAL atlas	LOO	SVM	0.80	0.85	25.68	26.02	41%	36%	NA	24.98	23.29	43.07	NA	3T Phillips	7.6/3.7	NA	SCZ	I
Pinaya et al., 2016	143	83	Cortical thickness and volume	K Fold	DBN	0.76	0.64	37.12	35.49	34%	33%	NA	12.74	16.92	58.59	160.68	1.5 Siemens	3000/3.42	NA	SCZ	I UNIFESP Deep ○
Yun et al., 2016	27	27	Cortical thickness or surface area	TTS	SVM	0.92	0.91	22.48	24.07	66%	40%	327.47	22.81	18.81	73	7.2	3T Siements Magnetom Trio	670/1.89	14	FEP	I
Salvador et al., 2017	128	127	GMV/WMV	10F	SVM+many	0.65	0.3	41.5	39.8	42%	42%	824	16.9	21.4	72.6	220.8	1.5 GE Signa	2000/4	7626	Scz	I
Chin et al., 2018	57	28	VBM GMD HO atlas	TTS	SVM	0.92	0.74	41.3	41.3	33%	33%	200.4	9.82	8.63	37.9	88.56	3T Phillips Achieva	8400/3.3	64	SCZ	I
Dwyer et al., 2018	71	74	GMV	K Fold	SVM	0.7 (I) 0.63 (E)	0.66 (I) 0.75 (E)	33.9	35.8	20%	31%	346.3	11.9	15.2	NA	54	3T Siemens Tim Trio	2530/(1.64,3.5,5.36,7.22,9.08)	NA	SCZ	I/E MRN, Cobre
Monte-Rubio et al., 2018	72	74	WMV, GMV	K Fold	GP	0.63	0.62	38.16	35.82	19%	31%	NA	NA	NA	NA	NA	3T Siemens Tim Trio	2530/(1.64,3.5,5.36,7.22,9.08)	NA	SCZ	I Cobre
Pinaya et al., 2018	35	40	Cortical thickness and volume	S-10F	DA	0.53	0.65	25.5	26.7	26%	48%	NA	NA	NA	NA	NA	3T Siemens Magnetom	9.7/4, 40/3	104	SCZ	I NUSDAST and HCP Datasets Deep ○
Schwarz et al., 2019	66	66	VBM GMD and mean thickness, sum surface area, and volume	Leave One Site Out	SVM+many	0.65	0.77	33.4	32.9	30%	24%	NA	NA	NA	NA	NA	3T GE	NA	152	SCZ	E UNIBA, CIMH, HUBIN, Kasp, UIO Datasets Pooled ♦
Vyskovsky et al., 2019	52	52	GMD, local volume change	LOO	RSENN	0.63	0.68	23	23	0%	0%	NA	NA	NA	NA	NA	1.5T Siemens Symphony	1700/3.93	NA	FES	I BRNO Deep
Winterburn et al., 2019	50	50	VBM GMD or RAVENS map or	10F	SVM+many	0.65	0.61	25.8	23.8	38%	36%	NA	23.9	24.3	NA	8.775	3T GE	13.4/5.7	1068592	FEP	E INNN, NUSDAST,

			cortical thickness																		CAMH Datasets Pooled ♦
Xiao et al., 2019	163	163	Cortical thickness or surface area	10F	SVM	0.80	0.86	23.54	23.61	55%	47%	NA	18.73	24.71	95.58	12.21	3T GE Excite	8.5/3.4	136	DN-FES	I
Chang et al., 2020	88	44	GMV or WMV	TTS	EDNN	0.84 (I) 0.81 (E)	0.89 (I) 0.59 (E)	45.8	45.8	58.3%	58.3%	NA	NA	NA	72.3	273.6	3T Siement Magnetom tim Trio	2530/3.5	NA	SCZ	I/E Deep
Chen et al., 2020	34	34	GMV, WMV	LOO	SVM	0.74	0.69	36.85	39.53	20%	32%	NA	NA	NA	NA	NA	3T Siemens Tim Trio	2530/(1.64,3.5,5.36,7.22,9.08)	Varied	SCZ	I Cobre
Nemoto et al., 2020	129	404	Cortical regional volume	TTS	LDA	0.6 (I) 0.63 (E)	0.81 (I) 0.75 (E)	36.5	35.5	39%	54%	547.8	NA	NA	NA	141.6	3T GE Signa Excite	4200/12.6	NA	SCZ	I/E Osaka ABC, Tokyo, Toyama, Kyushu Datasets Pooled ♦ °
Vieira et al., 2020	110	110	GMV and VBCT or cortical thickness and surface based volume	10F	DNN	0.59 (I) 0.7 (E)	0.60 (I) 0.38 (E)	28.5	29.7	38%	38%	NA	NA	NA	NA	13.2	3T	8.2/3.7	169	FEP	I/E Pelayo-Terán et al., 2008) Dataset Deep Pooled ♦
Yamamoto et al., 2020	50	51	VBM GMD	10F	SVM	0.61 (I) 0.62 (E)	0.82 (I) 0.83 (E)	38.8	36.5	48%	43%	NA	16	16.9	67.3	176.4	3T Siemens Verio	1900/2.48	NA	SCZ	I/E Nagoya and Toyama Dataset Pooled ♦ °
Latha et al., 2021	112	84	Radiomic features	K Folds	Fuzzy SVM	0.91	0.89	38.64	35.82	33%	39%	NA	15.4	15.44	NA	NA	3T Siemens Tim Trio	2530/(1.64,3.5,5.36,7.22,9.08)	Varied	SSD	I NAMIC, Cobre Dataset Pooled
Zhou et al., 2021	49	34	Cortical thickness	Nested CV	SVM	0.73	0.65	16.02	16.32	51%	55%	NA	NA	NA	NA	6.24	3T GE Signa	8348/3272	68	SCZ	I °
Vyskovsky et al., 2022	52	52	GMD, local volume change, intensities	TTS	CNN	0.58	0.54	23	23	0%	0%	NA	NA	NA	NA	NA	1.5T Siemens Symphony	1700/3.93	NA	FES	I BRNO Deep
Zhu et al., 2022	10	28	PCA of GMV and cortical thickness	10F	SVM	0.7 (I)	0.79 (I)	31.44	29.66	40%	41%	698.3	16.47	20.38	NA	87.36	3T GE Discovery	8.46/3.25	554992	SCZ	I/E °

Supplemental Table 1. Structural MRI datasets Statistical method: SVM = Support vector machine. FEP=First Episode Psychosis. TTS = Train Test Split. FES = First Episode Schizophrenia. SCZ = Schizophrenia. CV = cross validation. DN-FES = drug naïve first episode schizophrenia. LOO = leave one out. S-10F = stratified 10 fold. EDNN: explainable deep neural network. TR/TE = repetition time/echo time. WMV = white matter volume. GMV = gray matter volume. VBM = voxel based morphometry. CNN = convolutional neural network. 10F = 10 fold, RAVENS = regional analysis of volumes examined in normalized space. TTS = train test split. GMD = gray matter densities. WMV or GMV = a model was trained on either white matter volume or gray matter volume. The resulting values are the average between each model. TBV = total brain volume as measured by the sum of total white and gray matter volume. WMV and GMV--AAL atlas = white matter and gray matter volumes using an ROI analysis from the AAL atlas. VBM GMD HO atlas = voxel based morphometry to calculate gray matter densities using the Harvard-Oxford cortical and subcortical atlas. “and” = all measures were included in the model. “or” = each measure was used to train a model. PCA = principal component analysis. DBM=deformation based morphometry. GP=gaussian process. RSENN = random subspace ensemble neural network classifier. DA = deep autoencoders. DBN = deep belief network model. E = external data used for testing. I = internal training folds used for testing. Deep = manuscripts that use some form of deep learning. Pooled = data was pooled together for model training. ♦ indicates the pooled dataset did not overlap and was used for the primary analysis. ° = manuscript was kept in the secondary analysis after outlier exclusion.

Study	Psychosis Sample (N)	Control Sample (N)	Imaging	CV	Statistical method	Sensitivity	Specificity	Age Psychosis	Age Controls	Gender Psychosis (%F)	Gender Controls (%F)	CPZ Equivalent	PANSS Positive	PANSS Negative	PANSS Total	Illness Duration (months)	MRI Scanner	TR/TE (ms)	Feature Size	Addressing Motion	Diagnosis	Notes
Rathi et al., 2010	21	20	FA, MD	TTS	KNN	0.79 (0.69, 0.86)	0.78 (0.68, 0.85)	21.21	22.47	19%	25%	NA	NA	NA	NA	NA	3T	NA	NA	NA	FES	°
Ardekani et al., 2011	50	50	FA	TTS 50%	LDA	0.96 (0.96, 0.96)	0.96 (0.92, 1)	30.3	31.2	32%	32%	NA	NA	NA	NA	NA	1.5T GE	10000	13	NA	SCZ	Robinson et al., 2006 Dataset
Pettersson-Yeo et al., 2013	19	19	FA	LOO	SVM	0.68	0.63	22.42	23.32	52%	52%	4538.49	12.84	14	52.53	NA	3T GE Sigma	1300/104.5	5516	ECC, head motion, b-matrix reorientation and rejection of data outliers	FEP	°
Chen et al., 2018	60	60	Generalized FA	TTS	KNN	0.75	0.76	29.36	29.12	50%	50%	298.61	13.425	15.705	NA	276.3	3T Siemens Tim Trio	9600/130	76	ECC	SCZ	°
Mikolas et al., 2018	77	77	FA	LOO	SVM	0.60	0.65	28.51	28.32	44%	44%	337	13.9	15.7	62.4	3	3T Siemens	8300/84	129154	Visually inspected for excessive motion	FES	ESO dataset °
Deng et al., 2019	65	60	FA, RD, AD, MD	TTS 20% with LOO	RF	0.72 (0.67, 0.76)	0.75 (0.75, 0.75)	27.6	27.2	50%	48%	338.9	9.69	11.55	45.20	14.57	3T Phillips Achieva	8300/86	35	removed motion and ECC	FES	°
Wang et al., 2020	28	30	FA	Kfold	SVM	0.59 (0.46 0.72)	0.78 (0.73, 0.83)	22.32	22.47	39%	60%	NA	14.25	9.39	NA	42.72	3T Siemens SKYRA	6400/86	5516	ECC and motion distortion	SOC	°
Huang et al., 2020	18	19	FA	LOO	NSEAL	0.67	0.71	31.4	33.9	44%	31%	NA	NA	NA	NA	NA	3T	11000/76	Varied	ECC	SCZ	Deep °
Morgan et al., 2021	65	59	FA/MD	200fold	GPLC	0.54 (0.53, 0.54)	0.78 (0.77, 0.79)	27.9	29.2	33%	61%	NA	9.4	10.3	NA	NA	3T Siemens	NA	NA	Corrected movement, ECC, and susceptibility distortions	SCZ	Maastricht GROUP Dataset Pooled

Supplemental Table 2. DTI datasets Statistical method: SVM = Support vector machine. LDA= linear discriminant analysis. RF = random forest. KNN = K nearest neighbors. TTS = Train Test Split. TBSS = tract-based spatial statistics. FA = fractional anisotropy, AD = axial diffusivity, MD = mean diffusivity, RD = radial diffusivity. EPI = Echo Planar Imaging, FES = First Episode Schizophrenia, FEP = First Episode Psychosis, SCZ= Schizophrenia, LOO = leave one out TTS = train test split. SOC = schizophrenia with obsessive compulsive disorder comorbidity. CV = cross validation. ECC = eddy current correction. NSEAL = node level structure embedding and alignment learning. GPLC = gaussian process linear covariance model. All DTI studies only used internal training folds for model prediction. Deep = manuscripts that use a form of deep learning. Pooled = data was pooled together for model training. ♦ indicates the pooled dataset did not overlap and was used for the primary analysis. ° = manuscript was kept in the secondary analysis after outlier exclusion.

Study	Psychosis Sample (N)	Control Sample (N)	CV	Statistical method	Sensitivity	Specificity	Age Psychosis	Age Controls	Gender Psychosis (%F)	Gender Controls (%F)	CPZ Equivalent	PANSS Positive	PANSS Negative	PANSS Total	Illness Duration (months)	MRI Scanner	TR/TE (ms)	Feature Size	Minutes Rest	Diagnosis	Notes
Tang et al., 2012	22	22	LOO	PCA SVM	0.86	1	24.54	26.09	31%	31%	NA	NA	NA	75.64	38.52	1.5 T GE Signa	2000/40	4005	6	SCZ	I<br>○
Arbabshirani et al., 2013	28	28	TTS 40%	KNN	0.73	0.88	NA	NA	NA	NA	NA	NA	NA	NA	NA	3T Siemens Allegra	1500/27	36	5	SCZ	I<br>Deep<br>○
Fekete et al., 2013	8	10	LOO	SVM+many	0.85	0.9	44.1	41.8	0	0	NA	NA	NA	NA	NA	3T GE	3000/30	Varied	10	SCZ	I<br>NAMIC
Hu et al., 2013	10	10	LOO	SVM+many	0.75	0.8	44.1	41.8	0	0	NA	NA	NA	NA	NA	3T GE	3000/30	63903	10	SCZ	I<br>NAMIC
Su et al., 2013	32	32	LOO	SVM	0.82	0.78	24	25.01	22%	28%	NA	NA	NA	80.06	NA	1.5T GE Signa	2000/40	6670	6	SCZ	I<br>○
Yu et al., 2013a	32	38	OAR	SVM	0.93	0.91	24	24.44	21%	28%	NA	NA	NA	80.06	289.2	1.5T GE Signa	2000/45	6670	6	SCZ	I
Yu et al., 2013b	24	22	OAR	SVM	0.76	0.88	25.36	25.48	50%	50%	NA	21.8	22.38	87.14	NA	1.5T GE Signa	2000/40	6670	6	SCZ	I<br>○
Chyzhyk et al., 2015	40	28	10 Fold	SVM	0.61	0.65	38.50	39	41%	39%	523	NA	NA	NA	192	3T Siemens Trio	2500/24	10000	10	SCZ	Shinn et al 2013 Dataset
Kaufmann et al., 2015	71	196	LOO	LDA	0.42	0.92	28.2	31.5	41%	42%	NA	13.3	14.8	58.1	NA	3T GE Signa	2638/30	1081	9	SCZ	I<br>○
Kim et al., 2016	50	50	K Fold	DNN	0.74	0.78	35.94	35.5	14%	32%	NA	14.36	15	58.78	180	3T Siemens Trio	2000/29	6670	5	SCZ	I<br>Cobre<br>Deep
Rashid et al., 2016	60	61	10 Fold	SVM	0.81	0.68	35.85	35.44	22%	46%	NA	NA	NA	NA	NA	3T Siemens Allegra	1500/27	1176	5	SCZ	I<br>○
Chen et al., 2017	35	31	LOO	SVM	0.80	0.87	15.6	15.4	43%	58%	0	20.4	20.9	74.6	<2yrs	3T Siemens Tim-Trio	2000/30	NA	8	DN- FES	I<br>○
Guo et al., 2017	35	31	LOO	SVM	0.92	0.95	22.93	23.28	35%	50%	NA	22.68	21.18	88.11	24.14	3T Siemens Trio	2000/30	Varied	8	SCZ	I
Skatun et al., 2017	52	30	LOSO	LDA	0.80	0.75	30	27.3	37%	50%	NA	18.8	17.4	74.4	16.08	3T GE Signa	2000/27	1711	6	SCZ	E<br>Dataset KaSP, TOP, Hubin<br>Pooled ♦
Cui et al., 2018	56	55	10 Fold	SVM	0.88 (I)<br>0.78 (E)	0.76 (I)<br>0.81 (E)	23	32	46%	52%	NA	22	20	85	14	3T GE Discovery	2000/30	32	6	FES	I/E<br>○
Huang et al., 2018	41	23	LOO	SVM	0.90	0.65	23.51	25.61	46%	43%	NA	25.78	24.76	107.77	11.25	3T Siemens Magnetom Tim-Trio	2000/30	6670	10	FES	I<br>Data from Cui et al 2017<br>○
Kottaram et al., 2018	41	41	10 Fold	SVM	0.87	0.79	40.9	38.3	31%	41%	615.4	15.6	16.4	59.1	214.8	3T Siemens Tim-Trio	2000/40	4005	8	Treatment Resistant SCZ	I<br>○
Moghimi et al., 2018	82	88	Nested	SVM	0.60	0.75	31.35	31.75	30%	30%	NA	NA	NA	NA	NA	3T Siemens Trio	2000/30	1618	6	FES+SCZ	I
Zeng et al., 2018	474	607	LOO	SVM+many	0.79 (I)<br>0.76 (E)	0.82 (I)<br>0.76 (E)	NA	NA	NA	NA	NA	NA	NA	NA	NA	1.5-3T	Varied	Varied	Varied	SCZ	I/E<br>Xijing, AMU, Xiangya, Cobre, WUSTL<br>Pooled
Zhu et al., 2018	24	21	LOO	KDA	0.90	0.7	30.78	35.29	58%	71%	NA	NA	NA	97.83	NA	3T Siemens Tim-Trio	2000/30	NA	5	SCZ	I<br>Deep<br>○
Ji et al., 2019	230	185	LOO	KNN	0.8 (I)<br>0.74 (E)	0.72 (I)<br>0.67 (E)	NA	NA	NA	NA	NA	NA	NA	NA	NA	3T	Varied	35788	Varied	SCZ	I/E<br>AMU1 and 2, SWU, NKI, Cobre<br>Pooled
Jing et al., 2019	42	40	LOO	SVM	0.88 (I)<br>0.72 (E)	0.8 (I)<br>0.82 (E)	29.2	26.5	37%	52%	464.08	21.8	19.5	78.9	83.04	3T GE	2000/30	NA	8	SCZ	I/E<br>○
Matsubara et al., 2019	48	117	10 Fold	DNGM	0.52	0.79	36.2	31	22%	40%	NA	NA	NA	NA	NA	3T Siemens	2000/30	NA	5	SCZ	I<br>UCLA Dataset<br>Deep
Yan et al., 2019	558	542	K Fold	RNN	0.77 (I)<br>0.78 (E)	0.76 (I)<br>0.73 (E)	NA	NA	NA	NA	NA	NA	NA	NA	NA	3T	Varied	NA	Varied	SCZ	I/E<br>W. China Dataset<br>Deep
Yang et al., 2019	153	250	K Fold	CNE	0.64	0.82	NA	NA	NA	NA	NA	NA	NA	NA	NA	1.5-3T	Varied	Varied	Varied	SCZ	I<br>Cobre, UCLA, WUSTL<br>Deep<br>Pooled
Zhu et al., 2019	439	426	LOO	DAN	0.81	0.84	NA	NA	NA	NA	NA	NA	NA	NA	NA	1.5-3T	Varied	16200	Varied	SCZ	I<br>Huaxi, NBH, Cobre, Nottingham, Taiwan, Xiangya<br>Deep

																			Pooled
Lei et al., 2020	295	452	LOO	CNN	0.79	0.82	NA	NA	NA	NA	NA	NA	NA	NA	NA	1.5-3T	Varied	4005	Varied	SCZ	I<br>Cobre, UCLA, Maastrict, Dublin, W. China<br>Deep<br>Pooled
Lei et al., 2020b	295	452	K Fold	SVM	0.45	0.72	NA	NA	NA	NA	NA	NA	NA	NA	NA	1.5-3T	Varied	NA	Varied	Scz	I<br>Cobre, UCLA, Maastrict, Dublin, W. China<br>Deep<br>Pooled
Liu et al., 2020	38	38	LOO	SVM	0.74	0.91	26.3	25.4	50%	50%	NA	21.5	44.1	NA	32.64	3T Siemens Magnetom Tim-Trio	2000/30	19900	6.5	FES	I<br>°
Wang et al., 2020	60	71	K Fold	MCN	0.70	0.68	NA	NA	NA	NA	NA	NA	NA	NA	NA	3T Siemens Trio	2000/29	6670	5	SCZ	I<br>Cobre Dataset<br>Deep
Xiang et al., 2020	71	74	LOO	SVM	0.92	0.93	38.1	35.8	19.7%	31.1%	352.6	15	14.8	NA	NA	3T Siemens TIM	2000/29	NA	5	SCZ	I<br>Cobre Dataset
Yoshihara et al., 2020	114	175	LOO	SLR	0.72 (I)<br>0.71 (E)	0.79 (I)<br>0.71 (E)	NA	NA	NA	NA	NA	NA	NA	NA	NA	1-3T MRI systen	Varied	73	Varied	SSD	I/E<br>Testing<br>Cobre, TOP, JHU<br>(Training)<br>Kyoto dataset<br>°
Zhao et al., 2020	558	542	K Fold	GAN	0.73 (I)<br>0.71 (E)	0.72 (I<br>0.71 (E)	NA	NA	NA	NA	NA	NA	NA	NA	NA	3T	Varied	NA	Varied	SCZ	I/E<br>W. China Dataset<br>Deep
Zhu et al., 2020	72	75	K Fold	Forest	0.62	0.54	NA	NA	NA	NA	NA	NA	NA	NA	NA	3T	Varied	6786	Varied	SCZ	I<br>Cobre, UCLA
Gallos et al., 2021	72	74	K Fold	ML	0.57	0.73	NA	NA	NA	NA	NA	NA	NA	NA	NA	3T Siemens Trio	2000/29	NA	5	SCZ	I<br>Cobre<br>Deep
Lee et al., 2021	220	220	10 Fold	SVM	0.86	0.83	31.7	31.8	45%	50%	NA	15.1	17.4	66.33	112.8	3T GE	2500/30	NA	8	SCZ	I
Serin et al., 2021	12	15	10 Fold	Decision Tree	0.92	0.87	32.8	33.3	16%	6%	NA	NA	NA	NA	NA	1.5T GE Signa	2000/40	2701	17	SCZ	E<br>Dataset from Zalesky et al., 2010
Lei et al., 2022	505	907	LOO	CNN	0.74 (I)<br>0.73 (E)	0.97 (I)<br>0.67 (E)	NA	NA	NA	NA	NA	NA	NA	NA	NA	1.5-3T	Varied	6670	Varied	SCZ	I/E<br>Huaxi, UCLA, Dublin, Maastricht, Cobre<br>Deep<br>Pooled
Oh et al., 2022	171	161	10 Fold	CNN	0.83	0.83	34.38	33.73	47%	54%	449.33	13.69	11.57	50.05	77.7	3T Verio	2000/30	6670	5	SSD	I<br>Deep
Yuan et al., 2022	53	67	K Fold	SVM	0.64	0.55	36.7	34.8	20%	31%	NA	NA	NA	NA	NA	3T Siemens Trio	2000/29	6670	5	SCZ	I<br>Cobre

Supplemental Table 3. FC datasets Statistical method: SVM = Support vector machine. KNN = K nearest neighbors. FES = First Episode Schizophrenia, SCZ = Schizophrenia. SSD = Schizophrenia Spectrum Disorder. LOO = leave one out TTS = train test split. KDA= kernel discriminant analysis. GAN = generative adversarial network modeling. DN-FES = drug naïve first episode schizophrenia. PCA = principle component analysis. TR = repetition time TE = echo time. CV = cross validation. OAR = one against rest. LAAM = lattice auto-associative memories. TR = repetition time. TE = echo time. CNN = convolutional neural network. DNN = deep neural network. DAN = discriminant autoencoder network. RNN = recurrent neural network. DNGM = deep neural generative model. CNE = capsule network ensemble. MCN = multikernel capsule network model. ML = manifold learning. LOSO = leave one site out. NN= neural network. E = external data used for testing. I = internal training folds used for testing. JHU = Johns Hopkins first episode schizophrenia cohort. TOP = The outcome of Psychosis and Fitness Therapy, University Medical Centre. Utrecht. SLR = sparse logistic regression. Deep = manuscripts that use a form of deep learning. Pooled = data was pooled together for model training. ♦ indicates the pooled dataset did not overlap and was used for the primary analysis. ° = manuscript was kept in the secondary analysis after outlier exclusion.

Study	Psychosis Sample (N)	Control Sample (N)	Imaging	CV	Statistical Method	Sensitivity	Specificity	Age Psychosis	Age Controls	Gender Psychosis (%F)	Gender Controls (%F)	CPZ Equivalent	PANSS Positive	PANSS Negative	PANSS Total	Illness Duration (months)	MRI Scanner	T1 (TR/TE) (ms)	rsfMRI (TR/TE)	DTI (TR/TE)	Feature Size	Address Motion (DTI)	Minutes Rest	Diagnosis	Notes
Cabral et al., 2016	71	74	FC, GMV	K fold	SVM	0.78	0.71	38.1	35.8	19.7%	31.1%	352.6	15	14.8	NA	NA	3T Siemens TIM	2530/(1.64,3.5,5.36,7.22,9.0-8)	2000/29	NA	NA	NA	5	SCZ	I<br>Cobre Dataset
Qureshi et al., 2017	72	72	FC, cortical thickness, surface area, GMV, WMV	K fold	SVM	0.98	0.98	38.1	35.8	27%	19%	NA	14.96	14.53	58.71	108.36	3T Siemens TIM	2530/(1.64,3.5,5.36,7.22,9.0-8)	2000/29	NA	748	NA	5	SCZ	I<br>Cobre Dataset
Guo et al., 2018	161	168	FC, GMV, WMV, FA, MD, RD	10 Fold	SVM	0.85	0.86	44.99	43.17	59%	57%	NA	9.6	9.96	40.3	214.32	3T Siemens Magnetom Tim-Trio	3500/3.5	2500/27	11000/104	94	ECC	8	SCZ	I
Lee et al., 2018	47	23	FA, GMV, WMV	LOO	SVM	0.89	0.95	28.68	29.7	62%	65%	337.5	15.91	16.77	61.11	12.24	3T Philips Achieva	9/4.6	NA	5422/70	504	NA	NA	SCZ	I
Liang et al., 2019	54	48	FA, MD, GMV, cortical thickness, gyrification	K fold	Tree	0.72	0.78	24.8	25.25	66%	66%	0	NA	NA	93.49	17.89	3T Signa Excite	8.5/3.4	NA	10000/70.8	48	ECC	NA	FES	I<br>°
Qureshi et al., 2019	72	72	FC, ICA	K fold	CNN	0.98	0.97	38.1	35.8	27%	19%	NA	14.96	14.53	58.71	108.36	3T Siemens TIM	2530/(1.64,3.5,5.36,7.22,9.0-8)	2000/29	NA	Varied	NA	5	SCZ	I<br>Cobre Dataset<br>Deep
Zhuang et al., 2019	40	29	FA, MD, cortical thickness, GMV, surface area, mean curvature, curvature index, folding index	10 Fold	MK- SVM	0.94	0.66	27.13	27.03	45%	51%	0	NA	NA	73.54	6.51	3T Siemens Verio	2530/3.65	2000/30	10200/90	78489	ECC	6	DN-FES	I<br>°
Masoudi et al., 2020	64	81	FA, MD, MO, FC	K fold	CNN	0.99	0.97	38.92	37.98	20%	25%	NA	NA	NA	NA	NA	3T Siemens TIM	2530/(1.64,3.5,5.36,7.22,9.0-8)	2000/29	9000/84	Varied	ECC	5	SCZ	I<br>Cobre Dataset<br>Deep
Zhao et al., 2020a	135	148	FC, FA, GMV	10 fold	SVM	0.90	0.92	43.9	41.9	58%	56%	468.8	9.5	9.8	39.8	196.8	3T Siemens Magnetom Tim-Trio	3500/3.5	2500/27	11000/104	131	ECC	8	SCZ	I
Faria et al., 2021	87	62	FA, MD, GMV, WMV, FC	LOO	SIFA	0.80	0.45	22.5	23.4	28%	30%	381	NA	NA	NA	13.2	3T	6700/3.1	2000/30	8500/61	12309	Orientation and homogeneity correction	7	FEP	I<br>Dataset from Kamath et al., 2018<br>°
Lin et al., 2021	49	122	FA, FC, VBM GMD KSL	10 fold	MK-SVM	0.93 (I)<br>0.70 (E)	0.96 (I)<br>0.60 (E)	36.13	31.59	24%	46%	NA	NA	NA	NA	NA	3T Siemens Magnetom Tim-Trio	2530/3.44	2000/30	7000/92	60	ECC	8	SCZ	I/E<br>UCLA dataset
Rodrigue et al., 2021	414	288	FA, FC	TTS 20%	Ridge	0.51 (I)<br>0.47 (E)	0.52 (I)<br>0.47 (E)	35	37.1	42%	51%	NA	NA	NA	NA	NA	NA	NA	NA	NA	31145	Motion and ECC	NA	SCZ	I/E<br>Dataset ISMMS Olin BSNIP-1 BSNIP-2<br>Pooled ♦
Hu et al., 2022	148	76	GMV, WMV, CSF, FA, MD	10 Fold	CNN	0.86 (I)<br>0.64 (E)	0.56 (I)<br>0.63 (E)	32.72	31.33	31%	38%	NA	NA	NA	NA	NA	3T Philips Achieva	7200/3.2	NA	3725/63	NA	ECC reference to b0, inspection of signal dropout and artifacts. Exclusion of subjects with >3mm FD	NA	SCZ	I/E<br>IMH Dataset<br>Deep<br>°
Wang et al., 2022	79	205	FC, FA, GMV	10 fold	SVM	0.57	0.84	33.21	32.52	68%	53%	NA	22.47	23.22	85.23	>2years	3T Phillips Achieva	8.2/3.8	2000/30	6000/70	937	ECC	7	SCZ	I<br>°

Supplemental Table 4. Multimodal datasets Statistical method: SVM = Support vector machine. FES = first episode schizophrenia (no medication) DN-FES = drug naïve first episode schizophrenia. LOO = leave one out. TTS = train test split. SIFA = supervised integrated factor analysis. MK = multi kernel. FA = fractional anisotropy. MD = mean diffusivity. GMV = gray matter volume. WMV = white matter volume. RD = radial diffusivity. FEP = first episode psychosis. SCZ = schizophrenia. CV = cross validation. GMV = gray matter volume. VBM GMD KSL = voxel based morphometry gray matter density using Kullback-Leibler divergence-based similarity values. ECC = eddy current correction. CNN = convolutional neural network. ICA: independent component analysis. MO = mode of anisotropy. E = external data used for testing. I = internal training folds used for testing. Deep = manuscripts that use a form of deep learning. Pooled = data was pooled together for model training. ♦ indicates that pooled dataset did not overlap and was used for the primary analysis. ° = manuscript was kept in the secondary analysis after outlier exclusion.

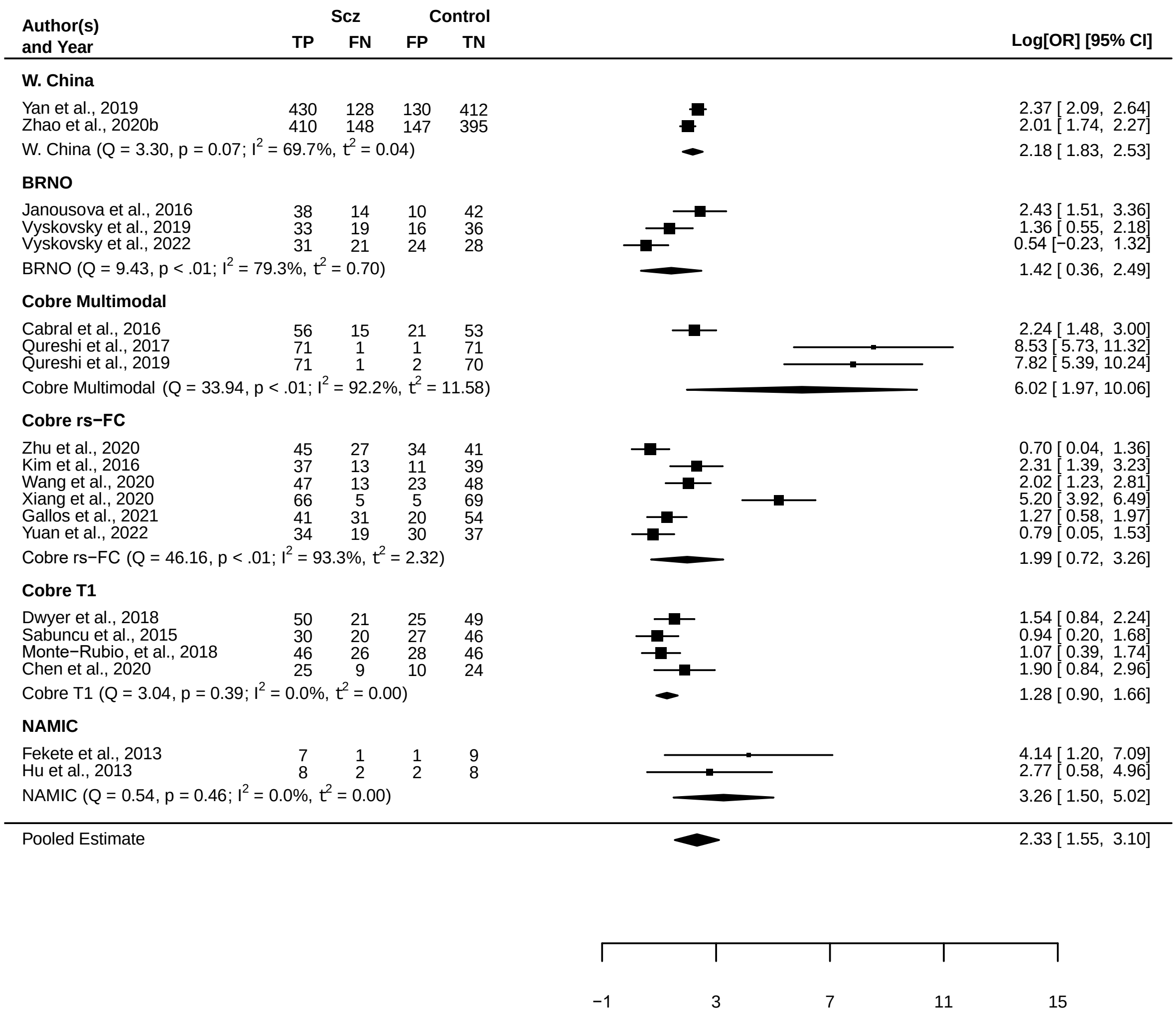

Supplemental Figure 1. Forest plot from studies used to construct a combined dataset score. To avoid potential type 1 errors with the main analysis. We calculated average classification scores using studies with the same dataset and imaging type. To highlight the range of classification scores we have grouped each study together.

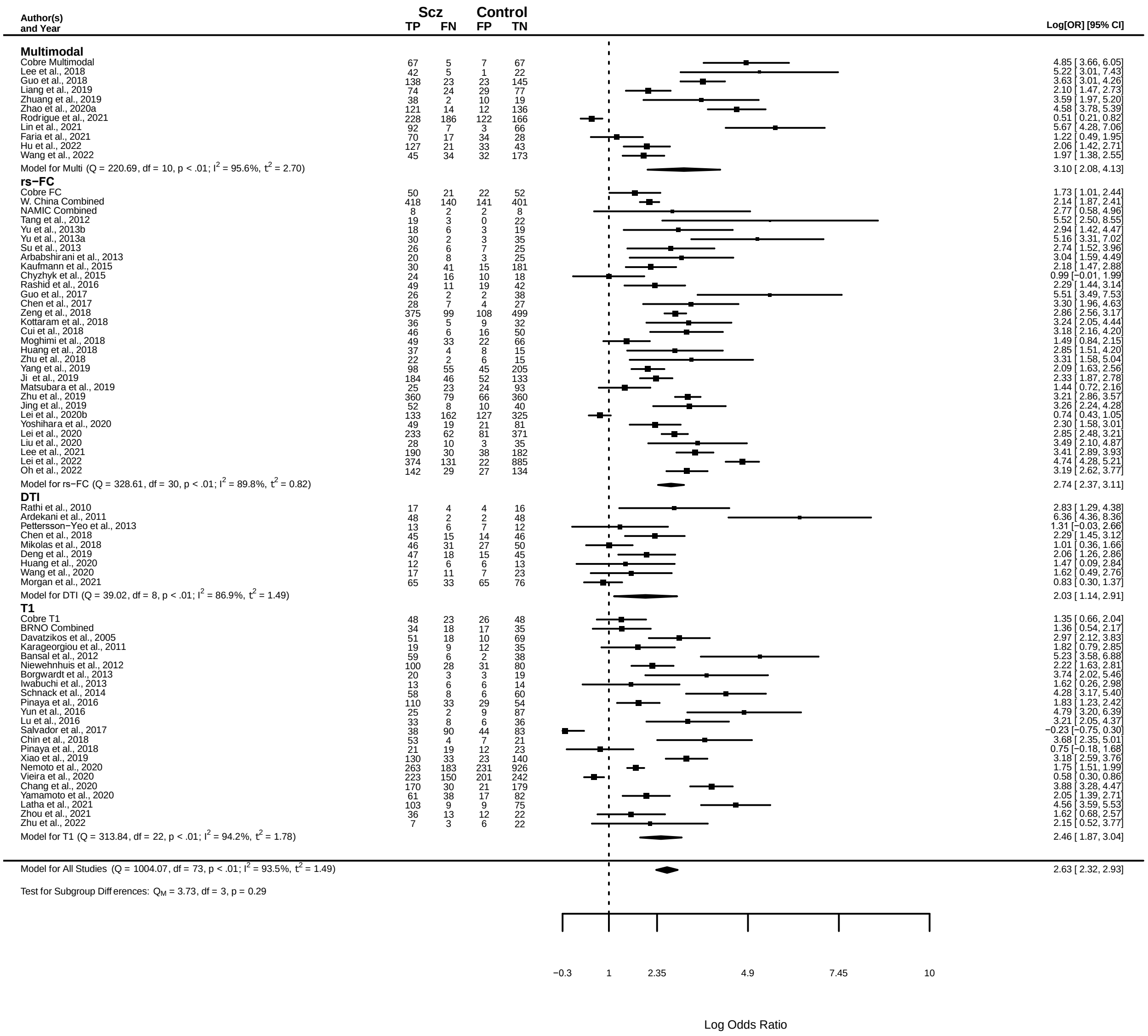

Supplemental Figure 2. Comparison of forest plot for log diagnostic odds ratio by imaging groups including studies that apply pooled datasets. Multimodal: classification that used at least two of the following: rs-FC, T1, and/or DTI as features. RS-FC: resting state functional connectivity. DTI: diffusion tensor imaging. T1: T1 weighted imaging. The point size of squares and polygons are a function of the precision of the estimates.

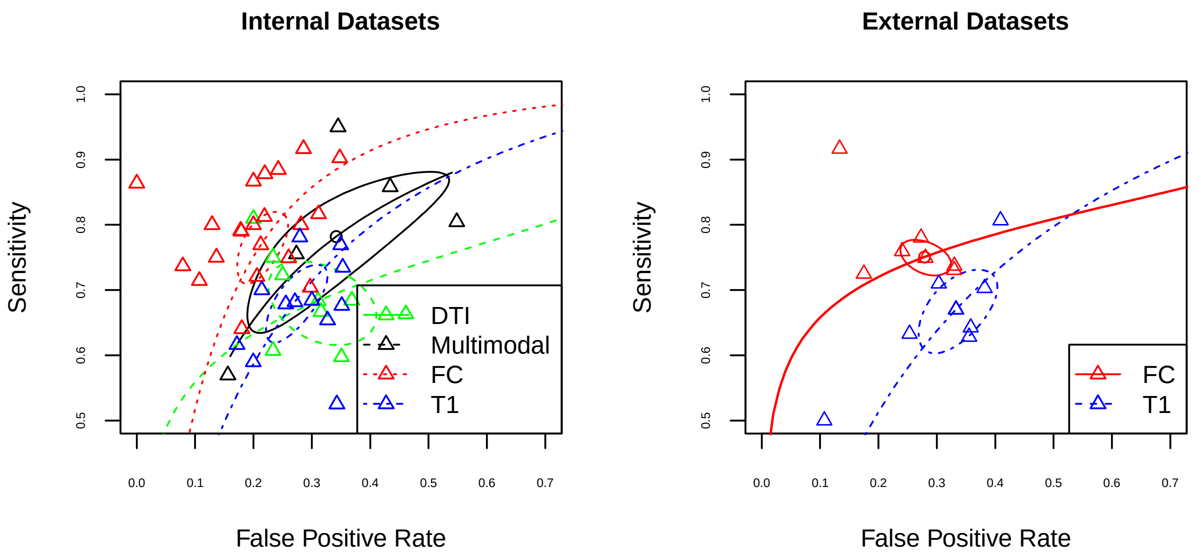

Supplemental Figure 3. Sensitivity plots of psychosis classification performance after the exclusion of outlier studies, when tested on independent internal (left; i.e., cross-validation) and external (right) datasets. In this case, large studies with pooled (repeated) datasets were included. Colors represent different imaging modalities; estimated summary receiver operating curves are shown overlaid for each of the imaging modalities with respective confidence interval regions surrounding mean sensitivity and specificity values. A significance difference was found between rs-FC (red) and DTI (green) when classification was tested using internal datasets (left). Within the external datasets (right), the rs-FC group provides a slight advantage relative to the T1 (blue) group.

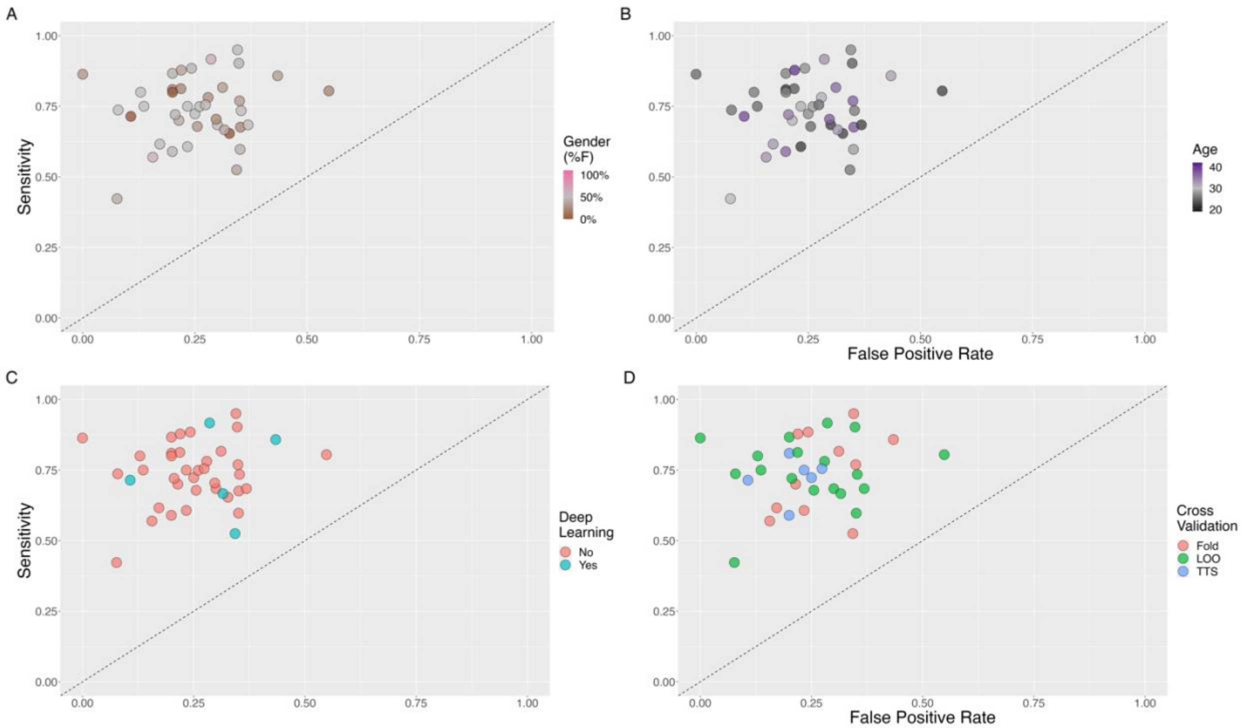

Supplemental Figure 4. Sensitivity plots of internal dataset psychosis classification performance and demographic variables after the exclusion of outlier studies, with results broken down by different demographic or analysis variables. Demographic plots are divided by (A) gender as represented via the percentage of females represented in the sample, (B) the mean age of the sample size. No obvious trends were seen for differences based on these two demographic characteristics. We also re-examined results based on (C) the method used for classification (contrasting simplified non deep and deep learning methods), and (D) the cross validation strategy used (e.g. Fold = K folds, LOO = leave one out, TTS = train test split). We again, did not find differences in classification performance based on these analysis variables.
